# Supplementary material for: Chromatin organization drives the search mechanism of nuclear factors
Source: Nat Commun. 2023 Oct 13;14:6433. doi: 10.1038/s41467-023-42133-5 (PMC10575952; doi:10.1038/s41467-023-42133-5)
Supplement: Supplementary file 1 — Supplementary Information [file 41467_2023_42133_MOESM1_ESM.pdf]

Supplementary Information for “Chromatin organization drives the search mechanism of nuclear factors”

Matteo Mazzocca†, Alessia Loffreda†, Emanuele Colombo, Tom Fillot, Daniela Gnani, Paola Falletta, Emanuele Monteleone, Serena Capozi, Edouard Bertrand, Gaelle Legube, Zeno Lavagnino, Carlo Tacchetti, and Davide Mazza*.

† Equally contributing authors.

* Corresponding author. To whom correspondence should be addressed. Email: mazza.davide@hsr.it

Content:

- 7 Supplementary figures.
- 2 Supplementary notes.
- 1 Supplementary table.
- Uncropped scans of western blots

Supplemental Figures

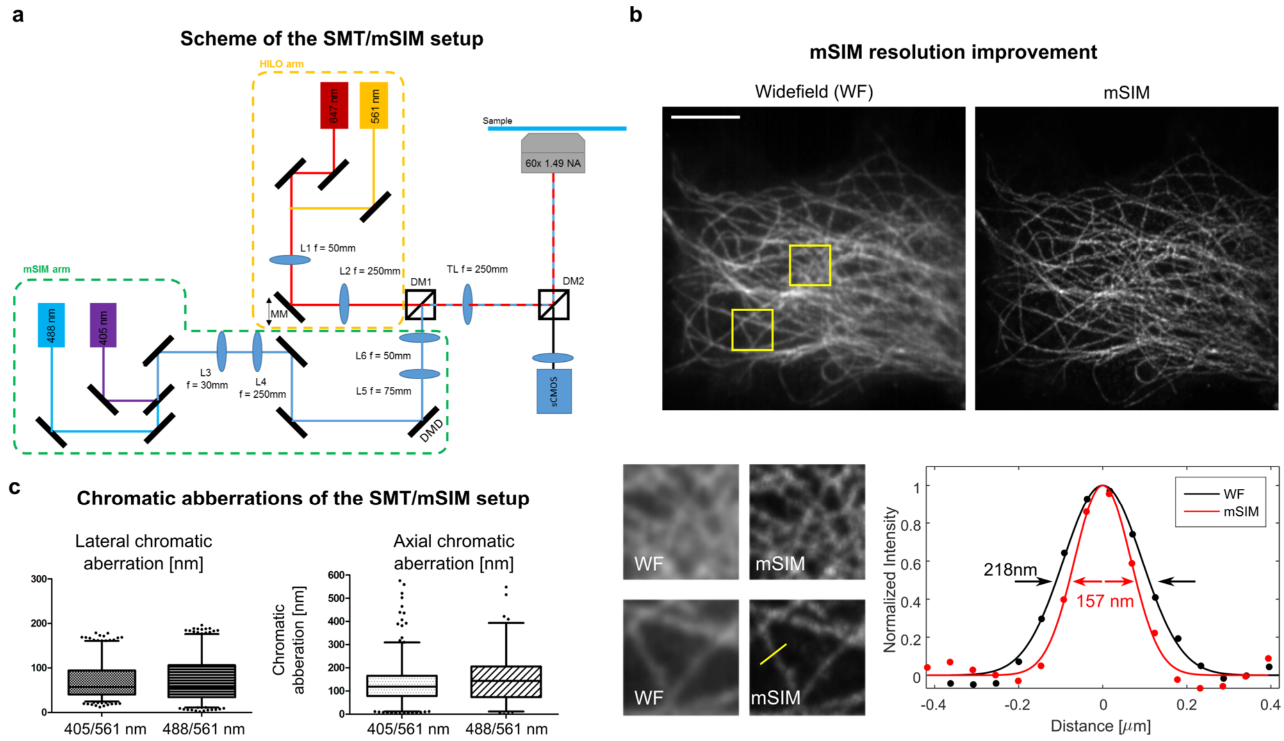

Supplementary Figure 1. Overview of the SMT/mSIM microscope. (**a**) Scheme of the optical setup. Two illumination arms allow for HILO and mSIM imaging of the same sample. The HILO illumination arm combines a 561nm and a 647 nm laser through a dichroic mirror, and projects a focused spot on a movable mirror (MM) that sits in a conjugated plane of the back focal plane of the microscope objective. Moving the movable mirror results in tilting the collimated beam in the front focal plane of the microscope objective. The mSIM illumination arm combines a 405 nm and a 488 nm laser through a dichroic mirror. The beam is then expanded by the couple of lenses L3 and L4 and directed to a digital micromirror device (DMD) that allows to project arbitrary patterns on the sample plane. The two illumination arms are combined through a dichroic mirror (DM1, Di03-R488-t3-25x36, Semrock Inc), and projected on the back aperture of the objective by an Olympus tube lens (TL). Emitted light by the sample is separated from excitation light by the dichroic mirror DM2 (Di03-R405/488/561/635-t3-25x36, Semrock inc.). The collected light is projected on a sCMOS camera (Orca Fusion, Hamamatsu Photonics). (**b**) The increase in lateral resolution of the mSIM microscope can be appreciated by imaging microtubules in fixed cells. Shown is the line profile along the segment highlighted in yellow (scale bar: 5µm). (**c**) We characterized chromatic aberrations between the HILO channel (561nm) and the mSIM channels (405 or 488 nm) by imaging 100nm microspheres (Tetraspeck Beads, ThermoFisher). Both lateral and axial chromatic aberrations were found to be below the resolution limit of mSIM. Source data are provided as a Source Data file.

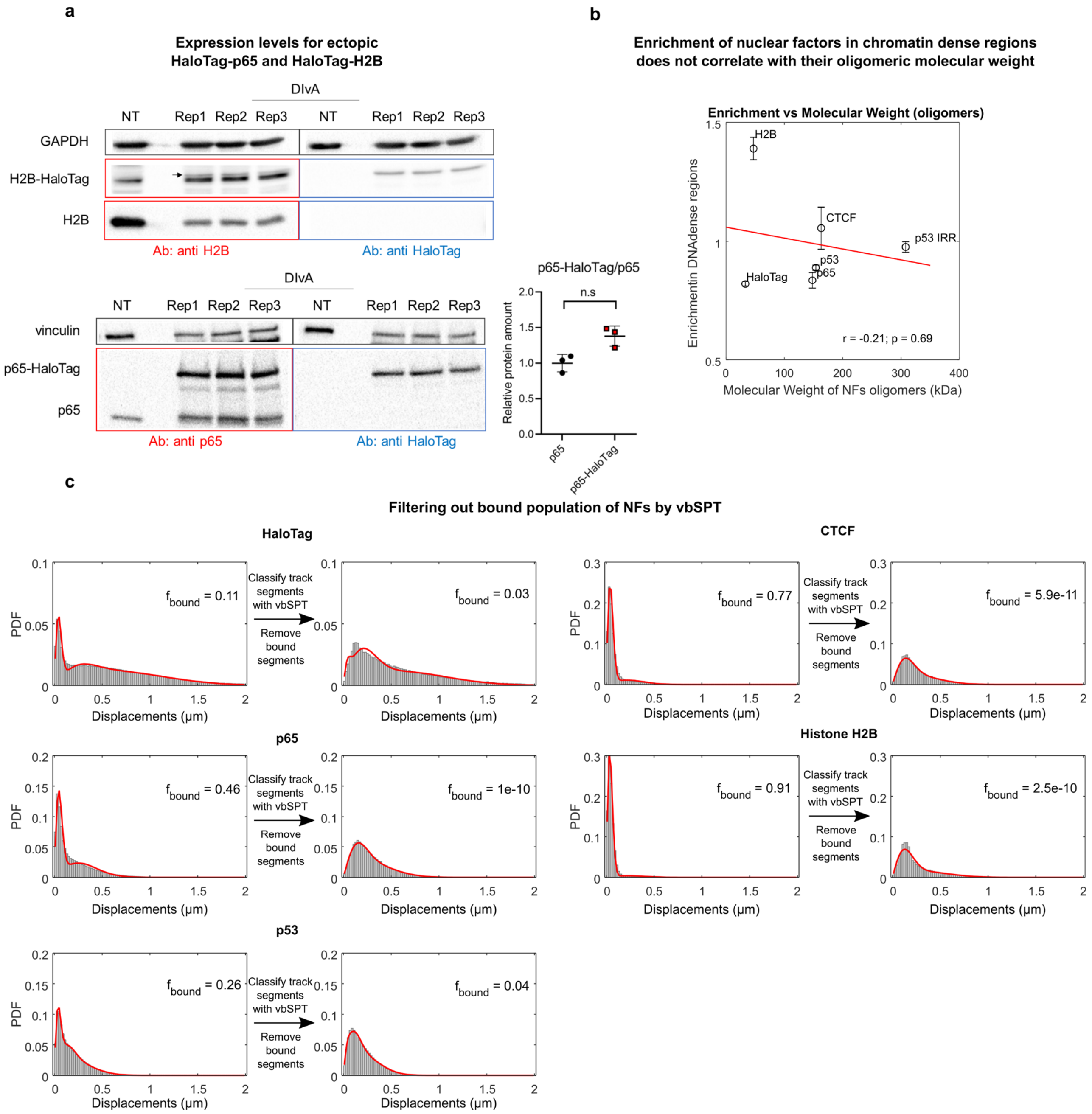

Supplementary Figure 2. Additional controls on NFs dynamics. (**a**) We quantified expression levels by western-blots for the two the two ectopically expressed factors, H2B-HaloTag (top) expression levels are very low compared to the endogenous H2B level (the H2B and the H2B-HaloTag signals shown on the western blot panel are obtained with different exposition times, 3.4 sec and 9.1 sec respectively. The arrow indicates the H2B-HaloTag band). p65-HaloTag is on average 1,4 fold more expressed than the endogenous p65 ($n_{replicates}$ = 3, statistical test Student's t-test, error bars: SD). (b) We evaluated the correlation between NFs molecular weight and enrichment in chromatin density, accounting for the expected oligomerization when they diffuse in the nucleus. Here HaloTag, H2B-HaloTag and CTCF-HaloTag are considered monomers, p65-Halotag is considered in complex with its partner p50 (untagged), p53-NT is considered dimeric (both tagged) and p53-IRR is considered tetrameric (all subunits tagged). No significant correlation is found between the two variables. Source data: same as fig. 1. (**c**) We use vbSPT to classify track segments into bound and diffusing components, and then filtered out the segments identified as bound molecules, in order to focus the analysis of diffusional anisotropy on the diffusing components only. To check that vbSPT successfully identified bound segments, we reanalyzed the distribution of displacements for each of the factors, after discarding those bound segments. In every case the residual bound fraction was estimated to be less than 5%. Source data (starting from data of fig. 2) are provided as Source Data file.

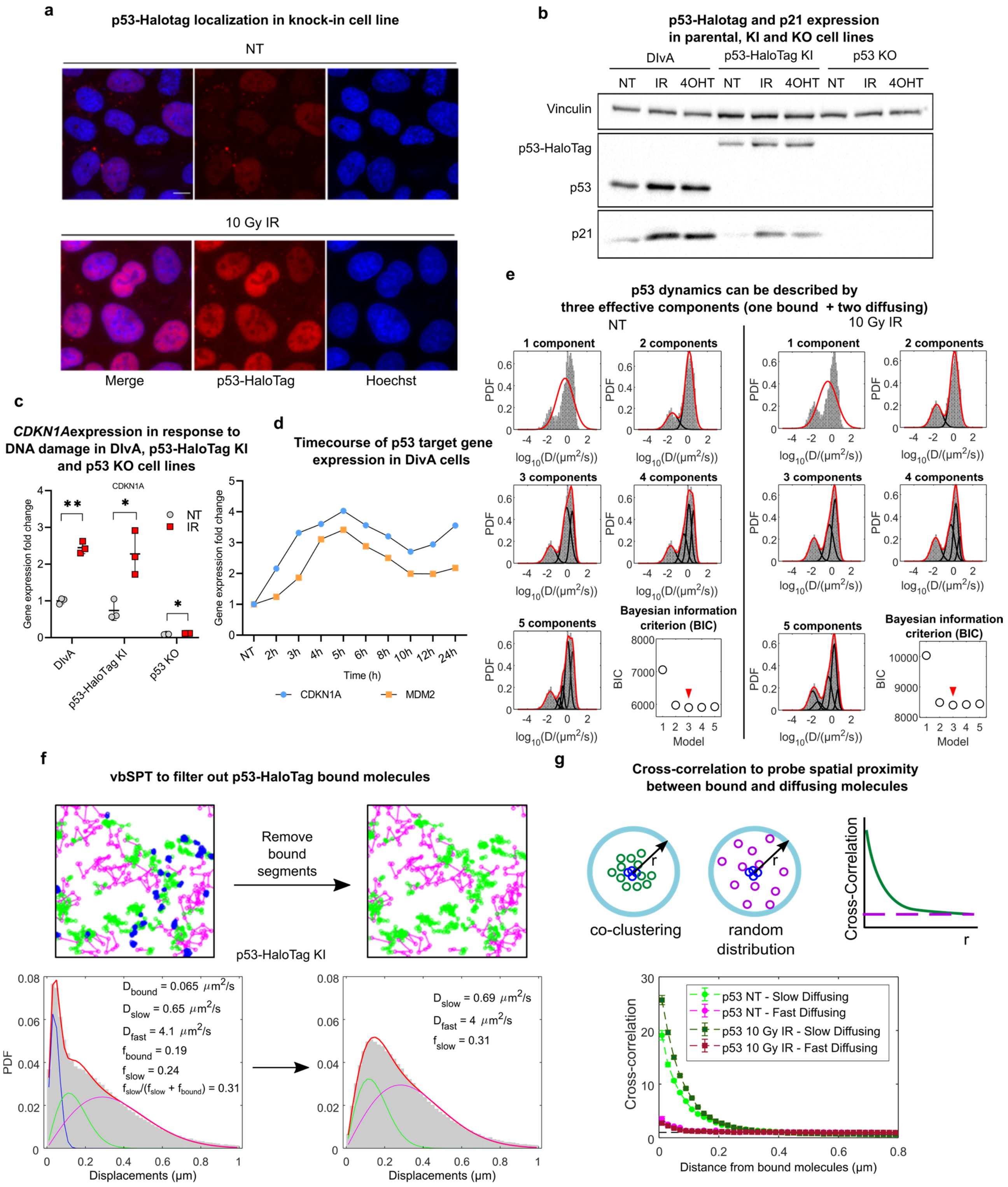

Supplementary Figure 3. Characterization of the p53-HaloTag knock-in (KI) cell line. (**a**) HaloTag-p53 displays the expected nuclear localization and accumulation in response to activation by 10-Gy IR. (**b**) Western-blot analysis reveals that the p53-HaloTag KI cell line displays accumulation of p53 and its target p21 upon activation by 10-Gy IR or 4OHT, while the p53-knock out (KO) cell line does not. (**c**) Analysis of p53 target gene expression by RT-qPCR displayed similar induction of p53 target genes in parental cells and p53-Halotag KI cells and no induction in p53-KO cells (error bars: SD, $n_{replicates} = 3$, statistical test: Student's t-test). (**d**) Time-course of target gene expression for *CDKN1A* and *MDM2* in p53-Halotag KI highlights a peak in p53 transcriptional activity between 4 and 5 hours post irradiation. (e) Model selection on diffusion coefficients extracted by mean-squared displacements analysis highlight that a model with three components (one bound + two diffusing) describes p53 mobility both in untreated conditions and upon 10-Gy IR. (**f**) vbSPT can be used to correctly filter out the bound population of p53 molecules. (**g**) Co-clustering of p53 bound and diffusing molecules by cross-correlation analysis. Slow diffusing p53 molecules co-cluster more frequently with bound molecules than fast ones. Source data (starting from data of fig. 3) are provided as Source Data file.

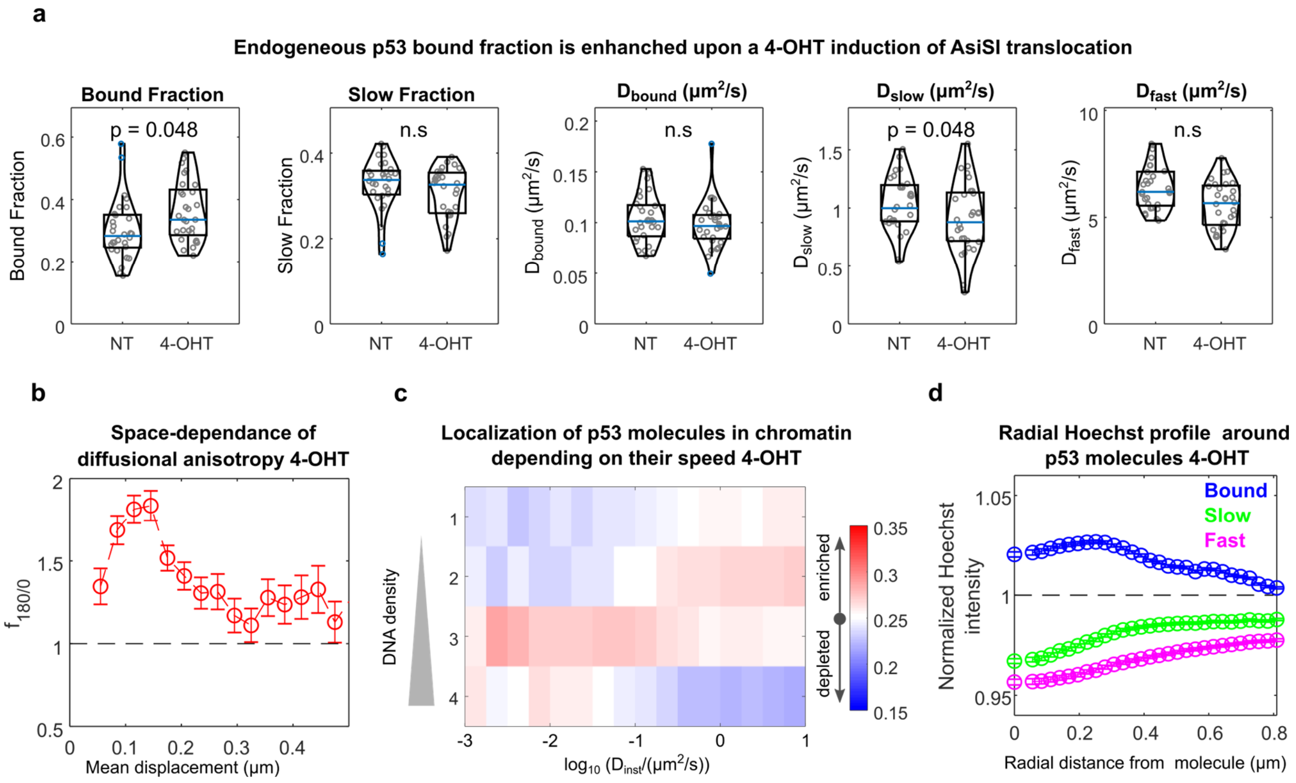

Supplementary Figure 4. Analysis of p53-HaloTag diffusion upon induction of DNA damage by AsiSI activation in DIvA p53-HaloTag knock-in cells. DIvA cells were treated with 4-OHT for 4 hrs to induce AsiSI translocation in the cell nucleus and analyzed with our SMT/mSIM pipeline. Induction of DNA damage by AsiSI results in increased p53 bound fraction (**a**) (n_{cells} = 29, 29 for untreated and 4-OHT respectively, statistical test Kolmogorov-Smirnov) and a diffusional anisotropy profile compatible with guided exploration (**b**) (error bars: s.e.m., evaluated by bootstrapping). Slow diffusing/bound p53 molecules localize in regions at higher DNA density than fast diffusing molecules, as evidenced by plotting p53 localization frequency in chromatin depending on their speed **(c)** and the radial Hoechst profile around p53 molecules (**d**). Source data are provided as Source Data file.

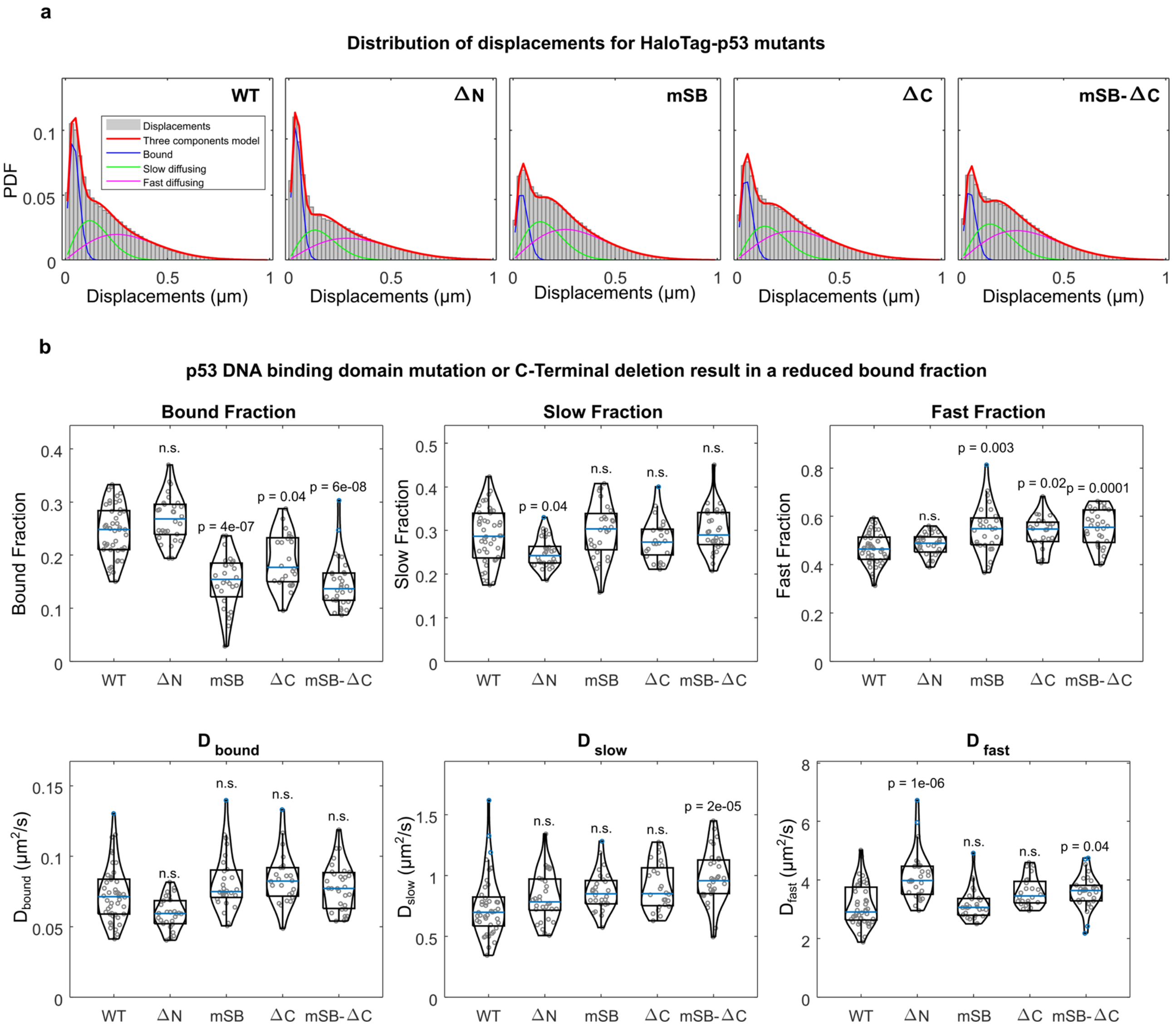

Supplementary Figure 5. paSMT analysis of p53 mutant dynamics. (**a**) Distribution of displacements for p53 WT and mutants. (**b**) Single-cell parameters extracted by fitting the distribution of displacements (statistical test Kruskal-Wallis, with Bonferroni correction for multiple testing). Source data (starting from data of fig. 5) are provided as Source Data file.

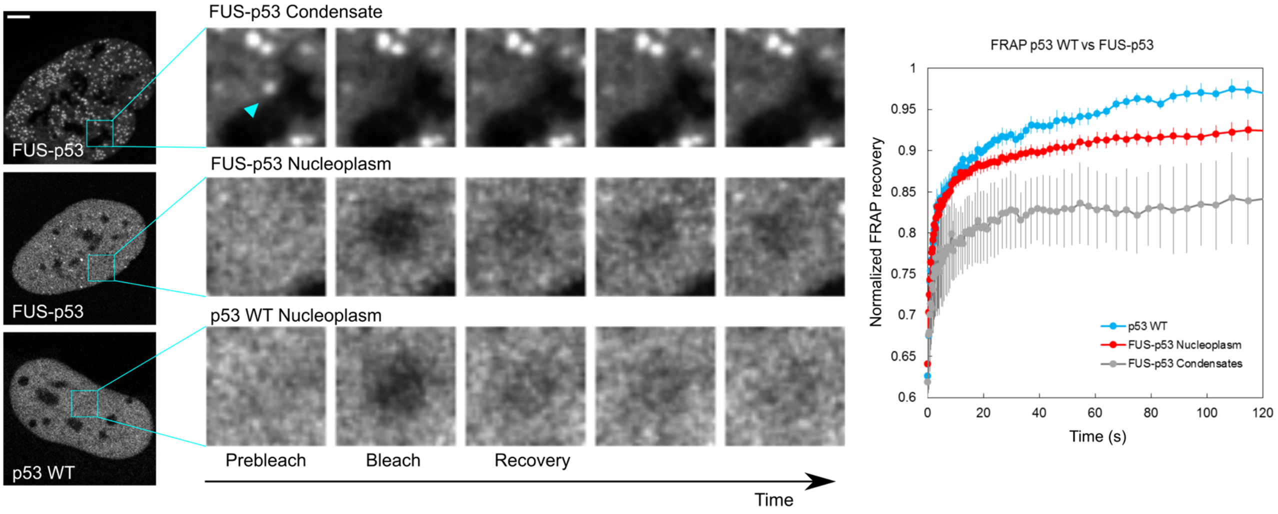

Supplementary Figure 6. FRAP analysis of p53 WT and FUS-p53 dynamics. FUS-p53 fluorescence recovery is slowed down compared to p53 WT, both in and outside condensates (error bars: s.e.m., n_{cells}= 9, 29, 5 for p53-WT, FUS-p53 nucleoplasm, FUS-p53 condensates respectively). Source data are provided as Source Data file.

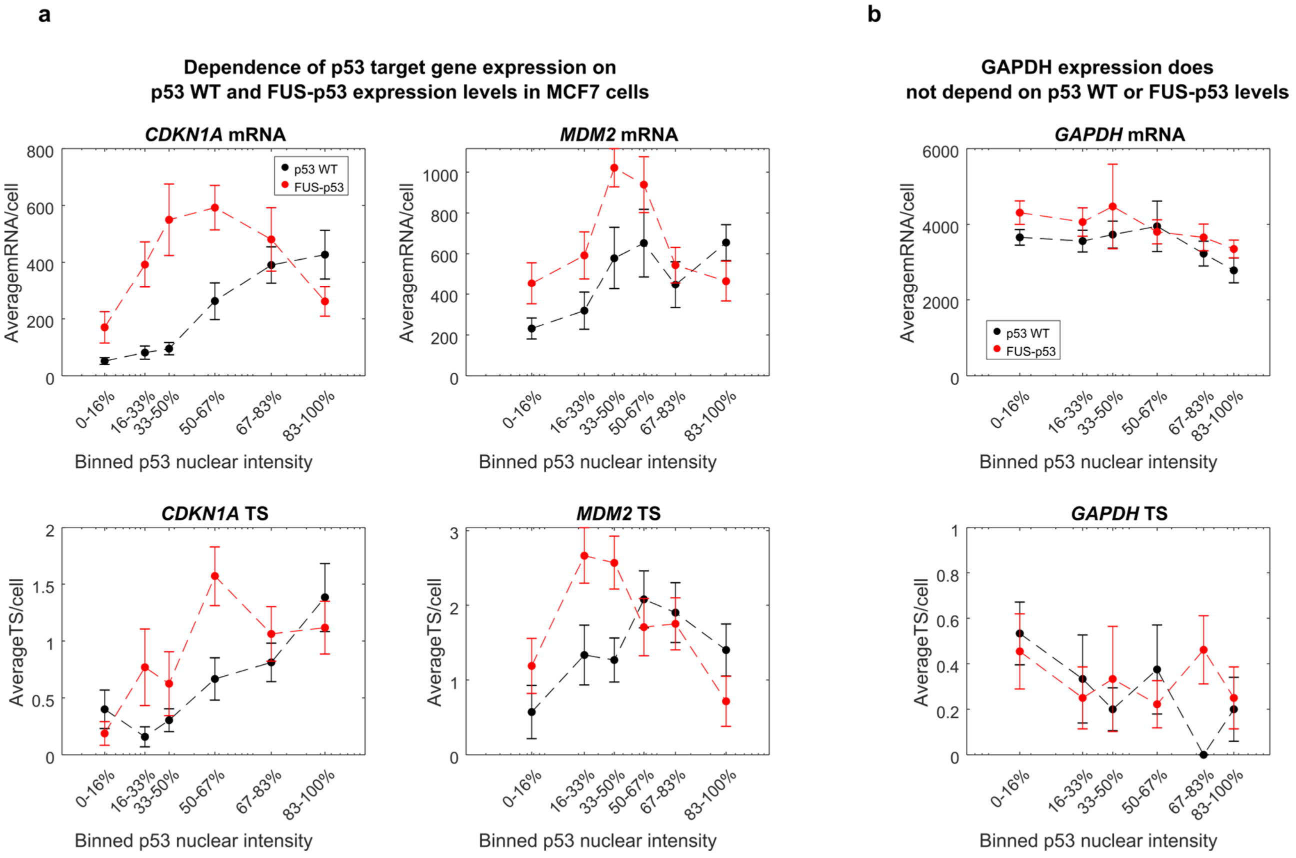

Supplementary Figure 7. Additional analysis on target gene expression dependence on FUS-p53 levels by smFISH. (**a**) Simultaneous imaging of HaloTag-p53 nuclear levels and mRNA expression by smFISH highlights that p53 target genes are activated in a bimodal manner by FUS-p53 also in breast cancer MCF7 cells. (**b**) The expression of the housekeeping gene GAPDH does not depend on p53 WT or FUS-p53 nuclear levels (error bars: s.e.m.). Source data are provided as Source Data file.

Supplementary Note 1: Reconstruction of mSIM images

As described by York et al.[1] in order to reconstruct a super-resolved and optically sectioned image from the 224 individual frames acquired in our mSIM set-up, it is necessary to: (i) identify the position of the illumination spots in the image plane at each acquisition frame; (ii) perform digital pinholing of the individual frames to get rid of out-of-focus blur; (iii) fuse together the images through pixel-reassignment that result in a $\sqrt{2}$ increase in the lateral resolution of the microscope. All these steps are performed with custom-written routines in Matlab.

Identification of the illumination spot position: The position at which illumination spots appears in the image, does not necessarily correspond to the points in which the DMD spots illuminate the sample, but rather they are the convolution product of the illumination pattern projected on the sample for the actual distribution of fluorescent labels in the sample. On average, however, the distance between the recorded spots should reflect the average distance between illumination points. Similar to what was performed in York et al.[1], we use this principle to find the lattice vectors that describe the 2D displacement between any two illumination spots. The position of all illumination points in the acquisition stack can be defined by two set of vectors (that need to be found for each individual acquisition): (i) the lattice vectors that describe the displacement between an illumination spot and the two nearest neighbor ones; (ii) the offset vectors, that specify the absolute position of the illumination spot closer to the top-left corner of the image in each of the images of the raw acquisition.

Identification of the lattice vectors. To identify the lattice vector we first localize the positions at which illumination spots appear in the sample by using the ThunderStorm plug-in[2] in ImageJ/FIJI. These coordinates are then used to generate a stack of binary images with ones at the pixels where the illumination spots have been localized and zero elsewhere. The images are then Fourier transformed and the Fourier magnitude images are then averaged together, giving rise to a periodic lattice of peaks, spaced by the inverse of the average spacing between peaks in the real images. Next, we search for peaks in the Fourier dimension, verify that we can find harmonics of the peaks found at lowest spatial frequency, and identify three candidate peaks with the lowest spatial frequency (that shows harmonics). We next verify that the vector sum and differences of the position vector of these identified peaks, also point to a detectable peak. If two vectors satisfy these conditions they are chosen as the lattice vectors in the Fourier space, and are then Fourier transformed to obtain real lattice vectors.

Identification of the offset vectors. Once we have the lattice vectors we can find the position of any other illumination spot in one of the images of the raw stack by knowing the position of one of them (i.e. by finding the position of the top left illumination spot in each them image, the offset vectors). To this scope we

translate the expected pattern of illumination over the x and y positions and maximize the autocorrelation between the expected illumination distribution and the experimental one.

Virtual Pinholing and pixel reassignment. To suppress out of focus blur, we next perform virtual pinholing, around the positions of illumination spots detected above. To this scope, we scale the raw images of a factor 10 in both dimensions, and we apply a gaussian mask of 130nm in standard deviation around the position of each detected illumination spot. Next, for every frame in the stack and for every spot position in the image the raw data from a squared region around the illumination spot are copied to the final image matrix in a squared region centered to the original coordinates multiplied by two. This procedure is analogous to move the information from each pixel to half of its distance from the illumination center, the principle on which Image Scanning Microscopy (ISM) is based[1]. Summing the result of this procedure for each illumination spot generates the final super-resolved image that is then scaled down by a factor 10 and deconvolved using the Richardson-Lucy algorithm.

1. York, A. G. *et al.* Resolution doubling in live, multicellular organisms via multifocal structured illumination microscopy. Nat Methods 9, 749–754 (2012).
2. Ovesný, M., Křížek, P., Borkovec, J., Švindrych Z., & Hagen, G. M. ThunderSTORM: a comprehensive ImageJ plugin for PALM and STORM data analysis and super-resolution imaging. Bioinformatics 30(16), 2389-2390 . (2014).

Supplemental Note 2: Correction of the distribution of displacements model for molecules going out of focus.

In SMT only molecules positioned within a slice of thickness of approximately $1\mu m$ around the focal plane can be localized and tracked. As a consequence, molecules diffusing with faster diffusion coefficients are more likely to diffuse out of focus, resulting in an underestimation of the fraction of molecules involved in this fast diffusion.

As described in the Methods section of the main text, we fit the distribution of displacements with a multi-component diffusion model, described by:

$$p(r)\Delta r = r\Delta r \sum_{i=1}^{3} \frac{f_i}{2D_i\Delta t} \exp\left(-\frac{r^2}{4D_i\Delta t}\right) p(\Delta z, D_i)$$

(Eq S.1)

Here $p(\Delta z, D_i)$, accounts for the probability of molecules of still staying in the slice with limits $-\Delta z$

to $+\Delta z$ around the focal plane. Considering that within this slice a single molecule can have any starting position z_s, $p(\Delta z, D_i)$ can be calculated as:

$$p(\Delta z, D) = \frac{1}{2\Delta z}\frac{1}{\sqrt{\pi}}\frac{1}{\sqrt{4D\Delta t}}\int_{-\Delta z}^{+\Delta z}\int_{-\Delta z}^{+\Delta z} e^{-\frac{(z-z_s)^2}{4D\Delta t}}\,\mathrm{d}z\,\mathrm{d}z_s$$

(Eq S.2)

By integrating over z, performing the substitution of variables $\theta = \frac{z-z_s}{\sqrt{4D\Delta t}} \rightarrow \mathrm{d}\theta = \frac{\mathrm{d}z}{\sqrt{4D\Delta t}}$, we obtain:

$$p(\Delta z, D) = \frac{1}{2\Delta z}\frac{1}{\sqrt{\pi}}\int_{-\Delta z}^{+\Delta z}\int_{\frac{-\Delta z-z_s}{\sqrt{4D\Delta t}}}^{\frac{+\Delta z-z_s}{\sqrt{4D\Delta t}}} e^{-\theta^2}\,\mathrm{d}\theta\,\mathrm{d}z_s = \frac{1}{2\Delta z}\int_{-\Delta z}^{+\Delta z} \mathrm{erf}\left(\frac{+\Delta z - z_s}{\sqrt{4D\Delta t}}\right) + \mathrm{erf}\left(\frac{+\Delta z + z_s}{\sqrt{4D\Delta t}}\right)\mathrm{d}z_s$$

Where we used the symmetric function $\mathrm{erf}(\theta) = 2/\pi \int_0^\theta e^{-\theta^2}\mathrm{d}\theta$

To solve this, we split the integral in two:

$$p(\Delta z, D) = \frac{1}{4\Delta z}\left[\int_{-\Delta z}^{+\Delta z} \mathrm{erf}\left(\frac{+\Delta z - z_s}{\sqrt{4D\Delta t}}\right)\mathrm{d}z_s + \int_{-\Delta z}^{+\Delta z} \mathrm{erf}\left(\frac{+\Delta z + z_s}{\sqrt{4D\Delta t}}\right)\mathrm{d}z_s\right]$$

In the first integral I we can substitute: $\theta = \frac{+\Delta z-z_s}{\sqrt{4D\Delta t}} \rightarrow d\theta = -\frac{dz_s}{\sqrt{4D\Delta t}}$ to yield:

$$I = -\sqrt{4D\Delta t}\int_{\frac{\Delta z}{\sqrt{D\Delta t}}}^{0} \mathrm{erf}(\theta)\,\mathrm{d}\theta = -\sqrt{4Dt}\left[\theta\,\mathrm{erf}(\theta) + \frac{e^{-\theta^2}}{\sqrt{\pi}}\right]_{\frac{\Delta z}{\sqrt{D\Delta t}}}^{0}$$

$$= 2\Delta z\,\mathrm{erf}\left(\frac{\Delta z}{\sqrt{D\Delta t}}\right) + \sqrt{\frac{4D\Delta t}{\pi}}\,e^{-\frac{(\Delta z)^2}{D\Delta t}} - \sqrt{\frac{4D\Delta t}{\pi}}$$

Similar, in the second integral, we substitute: $\theta = \frac{+\Delta z + z_s}{\sqrt{4D\Delta t}} \rightarrow d\theta = +\frac{dz_s}{\sqrt{4D\Delta t}}$

$$II = \sqrt{4D\Delta t}\int_{0}^{\frac{\Delta z}{\sqrt{D\Delta t}}} \mathrm{erf}(\theta)\,\mathrm{d}\theta = 2\Delta z\,\mathrm{erf}\left(\frac{\Delta z}{\sqrt{D\Delta t}}\right) + \sqrt{\frac{4D\Delta t}{\pi}}\,e^{-\frac{(\Delta z)^2}{D\Delta t}} - \sqrt{\frac{4D\Delta t}{\pi}}$$

Summing up the terms for I and II, we obtain:

$$p(\Delta z, D) = \frac{1}{2\Delta z}\left(2\Delta z\,\mathrm{erf}\left(\frac{\Delta z}{\sqrt{D\Delta t}}\right) + \sqrt{\frac{4D\Delta t}{\pi}}\,e^{-\frac{(\Delta z)^2}{D\Delta t}} - \sqrt{\frac{4D\Delta t}{\pi}}\right)$$

Which is the final expression for $p(\Delta z, D)$ used in Eq. S1.

Supplemental Tables.

smRNA FISH probes for ***CDKN1A*** (*HuluFISH,* PixelBiotech GmbH)	
Name	Sequence
CDKN1A_211	CTGCCGCAGAAACACCTGT
CDKN1A_0	CCCAGCCGGTTCTGACAT
CDKN1A_18	GCATGGGTTCTGACGGACATC
CDKN1A_85	ATCACAGTCGCGGCTCAGCT
CDKN1A_160	TCCAGTGGTGTCTCGGTGA
CDKN1A_219	CGTGGGAAGGTAGAGCTTGGG
CDKN1A_255	TCCTCCCAACTCATCCCG
CDKN1A_305	TCTTCCTCTGCTGTCCCCTGC
CDKN1A_345	GCGAGGCACAAGGGTACAAGA
CDKN1A_366	TTCAGCCTGCTCCCCTGA
CDKN1A_393	TGAGAGTCTCCAGGTCCACC
CDKN1A_427	TCTGTCATGCTGGTCTGCC
CDKN1A_447	CCGGCGTTTGGAGTGGTAGAA
CDKN1A_11	TTCCAGGACTGCAGGCTTCC
CDKN1A_41	ATGTAGAGCGGGCCTTTGAG
CDKN1A_121	GCCAGGGTATGTACATGAGGA
CDKN1A_187	CTCTCATTCAACCGCCTAGTT
CDKN1A_208	TGCCCAGCACTCTTAGGAAC
CDKN1A_251	ACACGGGATGAGGAGGCTTTA
CDKN1A_313	GGAGGAGGAAGTAGCTGGCAT
CDKN1A_335	TACCACCCAGCGGACAAGTG
CDKN1A_367	AGCGATGGGAAGGAGCCACAC
CDKN1A_393	GGGTGAATTTCATAACCGCCT
CDKN1A_414	GGTCTGAGTGTCCAGGAAAGG
CDKN1A_460	CCCTTCAAAGTGCCATCTGTT
CDKN1A_483	ATGATGCCCCCACTCGGTGAG
CDKN1A_530	CACCCTGCCCAACCTTAGAG
CDKN1A_550	GCTGTGCTCACTTCAGGGT
CDKN1A_576	TACCAGGTCCCCAGCTCA
CDKN1A_594	GGGTATCAAGAGCCAGGAGGG
CDKN1A_620	CCCCTGCCTTCACAAGACA
CDKN1A_676	TGCAGGTCAGAGGGGCCATGA
**smRNA FISH probes for *GAPDH*** (*HuluFISH,* PixelBiotech GmbH)	
GAPDH_35	CGGCTGGCGACGCAAAAGAAG
GAPDH_57	TGGTGTCTGAGCGATGTGGC
GAPDH_77	CGACCTTCACCTTCCCCA
GAPDH_102	GCCCAATACGACCAAATCCG
GAPDH_132	CCAGAGTTAAAAGCAGCCCTG
GAPDH_235	CGGTGCCATGGAATTTGCC
GAPDH_254	CTTCCCGTTCTCAGCCTTGA
GAPDH_291	TCCTGGAAGATGGTGATGGGA

GAPDH_317	CCCACTTGATTTTGGAGGGAT
GAPDH_345	TCCACGACGTACTCAGCGCCA
GAPDH_384	CCAGCCTTCTCCATGGTG
GAPDH_424	GGCAGAGATGATGACCCTTTT
GAPDH_452	TGACGAACATGGGGGCATCAG
GAPDH_499	GCTGATGATCTTGAGGCTGTT
GAPDH_530	TGCTAAGCAGTTGGTGGTGC
GAPDH_575	GTCCTTCCACGATACCAAAGT
GAPDH_601	GTGATGGCATGGACTGTGGT
GAPDH_629	GGGCCATCCACAGTCTTCT
GAPDH_648	TCACGCCACAGTTTCCCGGAG
GAPDH_674	TGATGTTCTGGAGAGCCCCGC
GAPDH_739	CTTCCCGTTCAGCTCAGG
GAPDH_786	CCACCACTGACACGTTGGCA
GAPDH_811	AGGTTTTTCTAGACGGCAGGT
GAPDH_841	CACCACCTTCTTGATGTCATC
GAPDH_888	TCAGTGTAGCCCAGGATGCCC
GAPDH_925	GGTGTCGCTGTTGAAGTCAGA
GAPDH_947	CAGCGTCAAAGGTGGAGGAGT
GAPDH_978	ACAAAGTGGTCGTTGAGGGCA
GAPDH_1018	GCTGTAGCCAAATTCGTTGTC
GAPDH_1061	ACTCCTTGGAGGCCATGTGGG
GAPDH_1107	TCTCTTCCTCTTGTGCTCTTG
GAPDH_1145	ACTGAGTGTGGCAGGGACTCC
**smRNA FISH probes for *MDM2*** (*Design Ready Stellaris,* Biosearch Technologies)	
MDM1	TGTTGGTATTGCACATTTGC
MDM2	TACAGCACCATCAGTAGGTA
MDM3	CGAAGCTGGAATCTGTGAGG
MDM4	TAAGTGTCTTTTTGTGCACC
MDM5	TAGTCATAATATACTGGCCA
MDM6	ATGTTGTTGCTTCTCATCAT
MDM7	TGGCACGCCAAACAAATCTC
MDM8	CTGTGCTCTTTCACAGAGAA
MDM9	TTCCTGTAGATCATGGTATA
MDM10	CTGCTGATTGACTACTACCA
MDM11	TGATCACTCCCACCTTCAAG
MDM12	GGTAGATGGTCTAGAAACCA
MDM13	CTAATTGCTCTCCTTCTAGA
MDM14	GTCGTTCACCAGATAATTCA
MDM15	CTATCAGATTTGTGGCGTTT
MDM16	TATTACACACAGAGCCAGGC
MDM17	TTCTTTCACAACATATCTCC
MDM18	CCTGTAGATTCACTGCTACT

MDM19	CACTTACACCAGCATCAAGA
MDM20	TCCAACCAATCACCTGAATG
MDM21	CTGATCTGAAACTGAATCCT
MDM22	CCTTCTTCACTAAGGCTATA
MDM23	CTGCCTGATACACAGTAACT
MDM24	ATTGCATGAAGTGCATTTCC
MDM25	AATGTGATGGAAGGGGGGGA
MDM26	GTGTTGAGTTTTCCAGTTTG
MDM27	ACTCTCTGGAATCATTCACT
MDM28	TCATCATTTTCCTCAACACA
MDM29	TGTGATTGTGAAGCTTGTGT
MDM30	GGCTGAGAATAGTCTTCACT
MDM31	ACTCTTTCACATCTTCTTGG
MDM32	AAGGGGCAAACTAGATTCCA
MDM33	TCACACAAGGTTCAATGGCA
MDM34	TAAGATGTCCTGTTTTGCCA
MDM35	TTTGCACATGTAAAGCAGGC
MDM36	TTGGTTGTCTACATACTGGG
MDM37	AGGGGAAATAAGTTAGCACA
MDM38	ATTCTCTTATAGACAGGTCA

Supplementary Table 1. List of Fluorescently labeled oligos used for smFISH

Uncropped scans of Western blots.

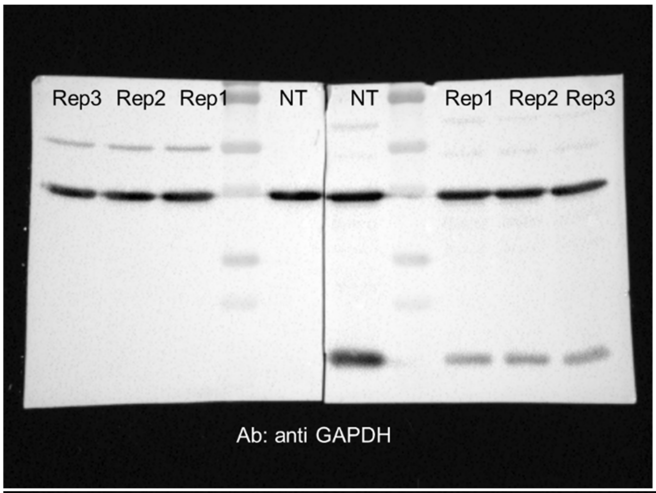

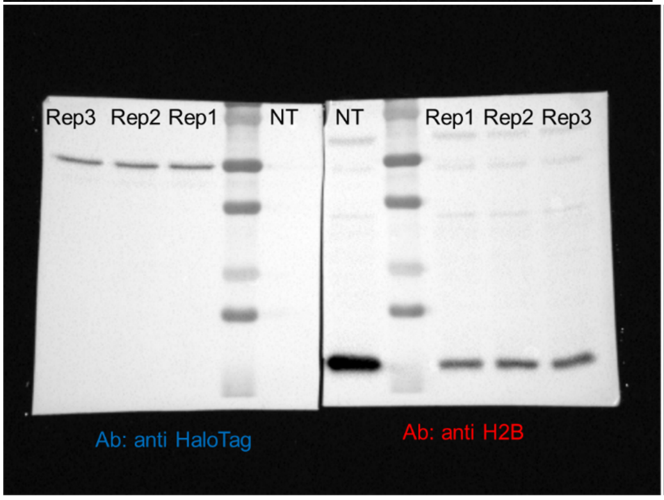

Exposure for Endogeneous H2B 3.4s

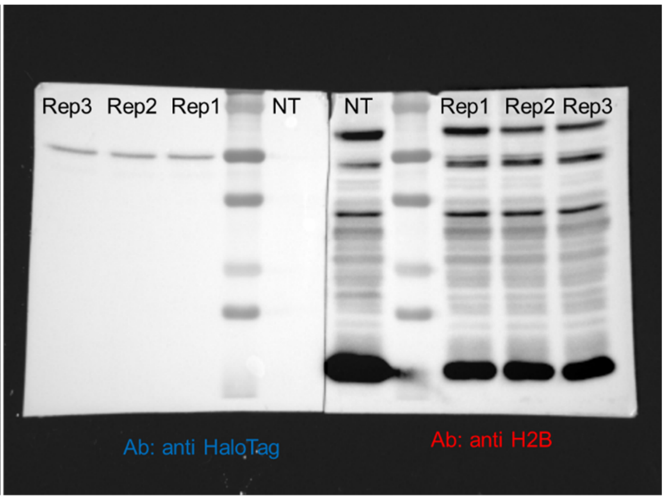

Exposure for HaloTag-H2B 9.1s

Gel in Supplementary Fig. 2a (H2B)

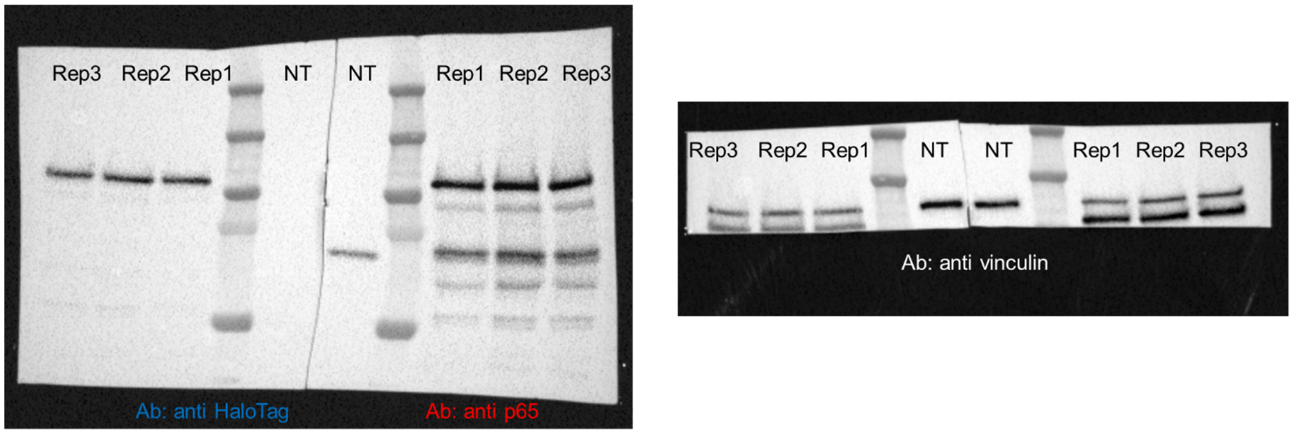

Gel in Supplementary Fig. 2A (p65)

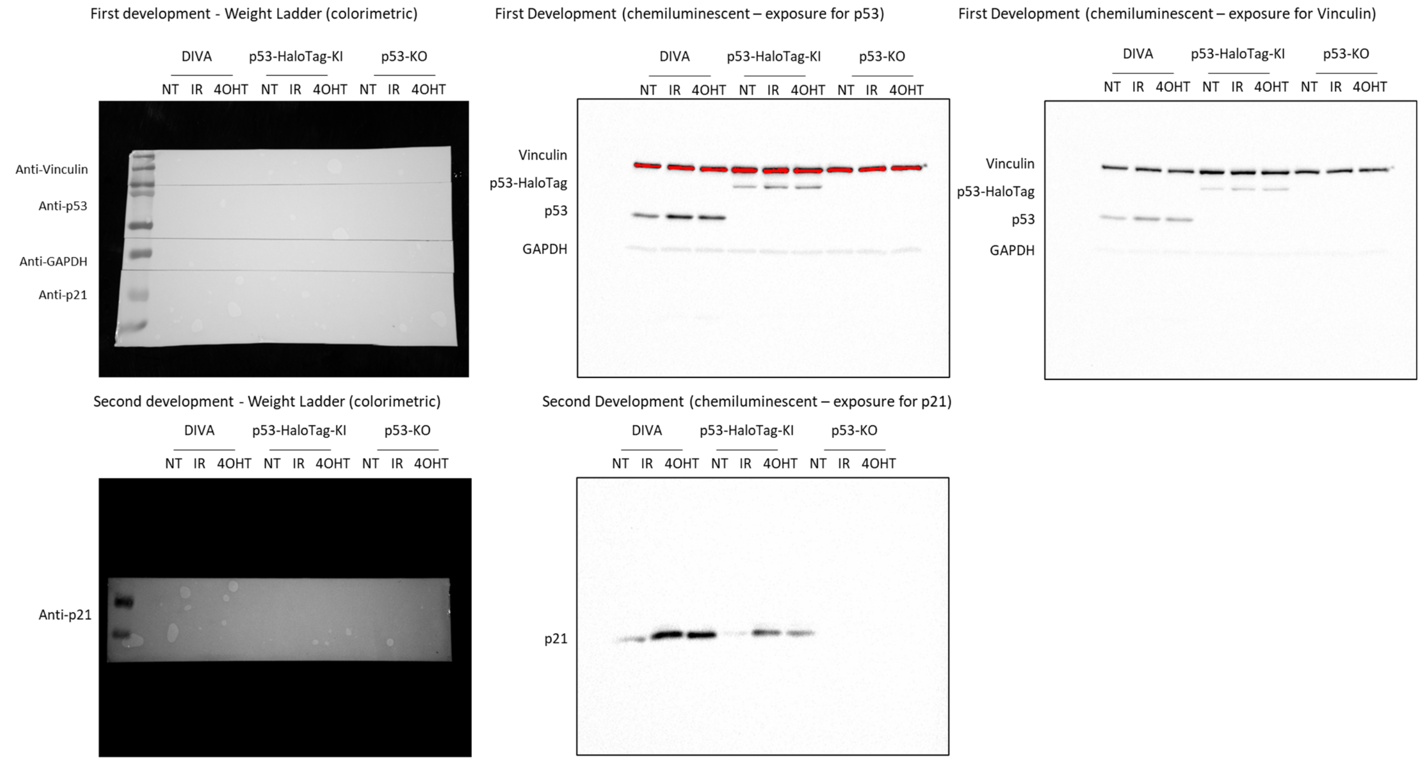

Gel in Supplemetary Fig. 3b
