# Supplementary material for: Chromatin organization drives the search mechanism of nuclear factors
Source: Nat Commun. 2023 Oct 13;14:6433. doi: 10.1038/s41467-023-42133-5 (PMC10575952; doi:10.1038/s41467-023-42133-5)
Supplement: Supplementary file 3 — Description of Additional Supplementary Files [file 41467_2023_42133_MOESM3_ESM.pdf]

Description of Additional Supplementary Files

File Name: Supplementary Movie 1
Description: Exemplary SMT/mSIM acquisition for Halotag, p65, p53, CTCF and H2B. Movies collected at 100fps and displayed at 30fps.

File Name: Supplementary Movie 2
Description: Exemplary paSMT acquisition for Halotag, p65, p53, CTCF and H2B. Movies collected at 100fps and displayed at 30fps.
