# Supplementary material for: Chromatin organization drives the search mechanism of nuclear factors
Source: Nat Commun. 2023 Oct 13;14:6433. doi: 10.1038/s41467-023-42133-5 (PMC10575952; doi:10.1038/s41467-023-42133-5)
Supplement: Supplementary file 6 — Reporting Summary [file 41467_2023_42133_MOESM6_ESM.pdf]

nature portfolio

Corresponding author(s): Davide Mazza

Last updated by author(s): Aug 29, 2023

Reporting Summary

Nature Portfolio wishes to improve the reproducibility of the work that we publish. This form provides structure for consistency and transparency in reporting. For further information on Nature Portfolio policies, see our Editorial Policies and the Editorial Policy Checklist.

Statistics

For all statistical analyses, confirm that the following items are present in the figure legend, table legend, main text, or Methods section.

n/a	Confirmed	
☐	☒	The exact sample size (*n*) for each experimental group/condition, given as a discrete number and unit of measurement
☐	☒	A statement on whether measurements were taken from distinct samples or whether the same sample was measured repeatedly
☐	☒	The statistical test(s) used AND whether they are one- or two-sided<br>*Only common tests should be described solely by name; describe more complex techniques in the Methods section.*
☒	☐	A description of all covariates tested
☐	☒	A description of any assumptions or corrections, such as tests of normality and adjustment for multiple comparisons
☐	☒	A full description of the statistical parameters including central tendency (e.g. means) or other basic estimates (e.g. regression coefficient) AND variation (e.g. standard deviation) or associated estimates of uncertainty (e.g. confidence intervals)
☐	☒	For null hypothesis testing, the test statistic (e.g. *F*, *t*, *r*) with confidence intervals, effect sizes, degrees of freedom and *P* value noted<br>*Give P values as exact values whenever suitable.*
☒	☐	For Bayesian analysis, information on the choice of priors and Markov chain Monte Carlo settings
☒	☐	For hierarchical and complex designs, identification of the appropriate level for tests and full reporting of outcomes
☒	☐	Estimates of effect sizes (e.g. Cohen's *d*, Pearson's *r*), indicating how they were calculated

Our web collection on statistics for biologists contains articles on many of the points above.

Software and code

Policy information about availability of computer code

Data collection	Micromanager for controlling the developed microscope.
Data analysis	Custom written Matlab Routines for reconstruction of the mSIM images v0.1 (Available here: https://github.com/shiner80/Recon_mSIM), using spot localizations obtained by the Fiji plugin Thunderstorm v1.3 (Ovesný et al., 2014, Bioinformatics). Single molecules were tracked with the ImageJ/Fiji plugin TrackMate v3.8.0 (Tinevez et al., 2017, Methods), smFISH data analyzed using the Matlab fishQUANT code build 9b64dc4b4eaf (Mueller et al., 2013, Nat Methods). HMM segmentation of single molecule trajectories performed using vbSPT (Persson et al., 2013, Nat Methods).

For manuscripts utilizing custom algorithms or software that are central to the research but not yet described in published literature, software must be made available to editors and reviewers. We strongly encourage code deposition in a community repository (e.g. GitHub). See the Nature Portfolio guidelines for submitting code & software for further information.

Data

Policy information about availability of data

All manuscripts must include a data availability statement. This statement should provide the following information, where applicable:

- Accession codes, unique identifiers, or web links for publicly available datasets
- A description of any restrictions on data availability
- For clinical datasets or third party data, please ensure that the statement adheres to our policy

Source data to generate all plots in main text and supplementary material are provided as a Source data file with this paper. The large volume of raw live-cell microscopy data (>1TB) generated for this work hampers sharing this dataset on free-of-charge generalist repositories. We therefore make the microscopy data available upon request.

Research involving human participants, their data, or biological material

Policy information about studies with human participants or human data. See also policy information about sex, gender (identity/presentation), and sexual orientation and race, ethnicity and racism.

Reporting on sex and gender	N/A
Reporting on race, ethnicity, or other socially relevant groupings	N/A
Population characteristics	N/A
Recruitment	N/A
Ethics oversight	N/A

Note that full information on the approval of the study protocol must also be provided in the manuscript.

Field-specific reporting

Please select the one below that is the best fit for your research. If you are not sure, read the appropriate sections before making your selection.

☒ Life sciences ☐ Behavioural & social sciences ☐ Ecological, evolutionary & environmental sciences

For a reference copy of the document with all sections, see nature.com/documents/nr-reporting-summary-flat.pdf

Life sciences study design

All studies must disclose on these points even when the disclosure is negative.

Sample size	SMT/mSIM and paSMT experiments: Based on our previous experience, we have evaluated that a sample size of 15 cells/conditions typically to identify differences > 10% across different samples/conditions. We aimed to repeat each experiment at least twice, leading to > 30 cells/ sample.<br>smFISH experiments: Based on previous experience on p53 target gene expression (Loffreda et al., Nat Comm. 2017) we aimed to collect ~10^2 cells per condition.<br>Western Blots/PCRs: We aimed to at least two replicates per condition.
Data exclusions	SMT/mSIM data discarded if cell moved during the acquisition.
Replication	Every SMT/mSIM experiment has been reproduced at least twice (Figures 1,4,5c,6f,h and associated supplementary figures) and paSMT experiment (Figures 2 , 3, 5b, 6d-e and associated supplementary figures) to verify reproducibility. Individual experiments provided consistent results. Data was then pooled together to provide comparisons at the single cell level.<br><br>Each in-situ Hybridization experiment (Figure 4c-d, Figure 5 d-f, Figure 6g and associated figures) were repeated twice. Individual experiments provided consistent results, but we show only one of the two replicates since technical differences in the efficiency of labelling of HaloTag-p53 inprevents comparison of HaloTag-p53 nuclear abundancies across experiments performed in different days.<br><br>FUS-p53 condensation (Figure 6b-c): Experiment performed multiple times that provided consistent results. Shown a single replicate.
Randomization	Not Applied, since all analysis performed automatically by software.
Blinding	Not Applied, since all analysis performed automatically by software.

Reporting for specific materials, systems and methods

We require information from authors about some types of materials, experimental systems and methods used in many studies. Here, indicate whether each material, system or method listed is relevant to your study. If you are not sure if a list item applies to your research, read the appropriate section before selecting a response.

Materials & experimental systems

n/a	Involved in the study
☐	☒ Antibodies
☐	☒ Eukaryotic cell lines
☒	☐ Palaeontology and archaeology
☒	☐ Animals and other organisms
☒	☐ Clinical data
☒	☐ Dual use research of concern
☒	☐ Plants

Methods

n/a	Involved in the study
☒	☐ ChIP-seq
☒	☐ Flow cytometry
☒	☐ MRI-based neuroimaging

Antibodies

Antibodies used

Mouse Monoclonal Anti-p53 (DO-1), Santa Cruz Biotechnology, Cat# sc-126, lot #B1221
Rabbit monoclonal anti-p21 [EPR362], Abcam; Cat# ab109520, lot # GR3255492-3
Rabbit monoclonal anti-GAPDH [EPR6256], Abcam, Cat# ab128915, Lot #GR32725
Mouse monoclonal anti-vinculin, Thermo-Fisher, Cat# MA5-11690, Lot #SA2317895
Anti-rabbit IgG, HRP-linked Antibody , Cell Signaling, Cat #7074, Lot# 28
Anti-mouse IgG, HRP-linked Antibody , Cell Signaling, Cat #7076, Lot# 36
Rabbit monoclonal anti-NF-κB p65, Cell Signaling cat. D14E12 XP®
Rabbit polyclonal anti-Histone H2B, Abcam cat. ab1790
Mouse monoclonal anti-HaloTag,Promega G921A

Validation

For each antibody we used in this work, the manufacturer provides tens to hundreds references. At these links there is the list of references for each antibody:
https://www.scbt.com/it/p/p53-antibody-do-1
https://www.abcam.com/products/primary-antibodies/p21-antibody-epr362-ab109520.html
https://www.abcam.com/products/primary-antibodies/gapdh-antibody-epr6256-loading-control-ab128915.html?productWallTab=Abreviews
https://www.thermofisher.com/antibody/product/Vinculin-Antibody-clone-VLN01-Monoclonal/MA5-11690
https://www.cellsignal.com/products/primary-antibodies/nf-kb-p65-d14e12-xp-rabbit-mab/8242
https://www.abcam.com/products/primary-antibodies/histone-h2b-antibody-chip-grade-ab1790.html
https://www.citeab.com/antibodies/7602044-g9211-anti-halotag-monoclonal-antibody

Eukaryotic cell lines

Policy information about cell lines and Sex and Gender in Research

Cell line source(s)

U-2 OS derived DIvA: Gaelle Legube Lab (Aymard et al., Nat. Struct. Mol. Biol., 2014)
U-2 OS CTCF-HaloTag knock-In: Robert Tijan Lab (Hansen et al. Nat Chem Biol, 2020).
DIvA p53-HaloTag knock-in, this paper
DIvA p53-HaloTag knock-out, this paper
MCF7 p53 knock-out: Our Lab (Loffreda et al. Nat Comm, 2017)

Authentication

U-2 OS derived DIvA cell line has been authenticated by probing the generation of AsiSI restriction enzyme dependant DBS, obtained upon OHT addition and looking at DBS with p-H2aX staining.
U-2 OS CTCF-HaloTag knock-In cell line has been verified by labeling the protein with an HaloTag fluorescent dye and looking at its localization by fluorescence microscopy and the amount of the protein by FACS analysis.
DIvA p53-HaloTag knock-in and Knock-out cell lines have been generated for this work and have been validated by both western blot and immunofluorescence analysis.
MCF7 p53 knock-out cells have been generated by our group and published in a previous work and the authentication was performed by western blot and immunofluorescence.

Mycoplasma contamination

All cell lines have been tested routinely and resulted negative for mycoplasma contamination

Commonly misidentified lines
(See ICLAC register)

No commonly misidentified lines as reported in the ICLAC register have been used in this study.
